# Letter to the Editor: “How Can Biomedical Engineers Help Empower Individuals With Intellectual Disabilities? The Potential Benefits and Challenges of AI Technologies to Support Inclusivity and Transform Lives”

**DOI:** 10.1109/JTEHM.2023.3331977

**Published:** 2023-11-09

**Authors:** Alessandro Di Nuovo

**Affiliations:** Department of ComputingSheffield Hallam University 7314 S1 1WB Sheffield U.K.; Advanced Wellbeing Research CentreSheffield Hallam University 7314 S1 1WB Sheffield U.K.

**Keywords:** Human-centered innovation, healthcare technologies, ethical AI, equity, inclusion, devices for dignity, socially assistive robotics

## Abstract

The rapid advancement of Artificial Intelligence (AI) is transforming healthcare and daily life, offering great opportunities but also posing ethical and societal challenges. To ensure AI benefits all individuals, including those with intellectual disabilities, the focus should be on adaptive technology that can adapt to the unique needs of the user. Biomedical engineers have an interdisciplinary background that helps them to lead multidisciplinary teams in the development of human-centered AI solutions. These solutions can personalize learning, enhance communication, and improve accessibility for individuals with intellectual disabilities. Furthermore, AI can aid in healthcare research, diagnostics, and therapy. The ethical use of AI in healthcare and the collaboration of AI with human expertise must be emphasized. Public funding for inclusive research is encouraged, promoting equity and economic growth while empowering those with intellectual disabilities in society.

## I. Introduction

Rapid technological advancements in Artificial Intelligence (AI) are revolutionizing healthcare and reshaping our daily lives. However, while they present exciting opportunities, they also pose critical ethical and societal challenges. Among the challenges, there is a pressing need to develop technologies that can autonomously adapt to diverse needs so that they are beneficial to all individuals, regardless of their background, abilities, or characteristics. Unfortunately, we often witness the opposite scenario, where users should adapt to the technology.

This situation has a negative impact on individuals with intellectual disabilities, who already face unique challenges in various aspects of their lives and can struggle more to adapt because of their condition. Consequently, individuals with intellectual disabilities not only are left behind and miss out on the benefits of the AI revolution, but this creates even larger gaps with the rest of the population.

Nevertheless, a Responsible Research and Innovation (RRI) approach can ensure that it both benefits and respects the rights and dignity of all individuals. In this regard, biomedical engineers possess the essential transdisciplinary knowledge and skills required to promote collaborative efforts within intersectoral partnerships and multidisciplinary teams to design, develop, and deploy human-centered AI technologies into the real world, serving as a powerful tool to bridge the gap and empower individuals with intellectual disabilities.

Among the many opportunities, AI technologies can reduce gaps by personalizing learning and skill development. Biomedical Engineers should prompt the advance of AI-powered educational platforms able to adapt to the specific needs and learning styles of individuals with intellectual disabilities, providing tailored lessons and resources. One such challenge is personalized learning, which can help individuals acquire essential life skills, enhancing their self-esteem and self-sufficiency. AI-driven communication tools improve accessibility and inclusivity. Predictive text and speech recognition algorithms need to be developed to enable individuals with communication difficulties to express themselves more effectively. AI-driven real-time captioning, voice assistants, and screen readers need to be developed to enhance accessibility for people with diverse needs. Smart home technology can favour independent living by assisting with tasks such as reminders for medication, meal preparation, and home security. AI-powered wearables and mobile apps can provide guidance and support for individuals in managing their daily routines on their own. Interestingly, one opportunity for biomedical engineers is to design more inclusive job environments with human-centred automation in the workplace, breaking down barriers to entry and allowing more people with intellectual disabilities to be fully integrated into the workforce while also challenging stigmas and biases.

Furthermore, AI technologies need to be harnessed to enable health researchers and caregivers analyse vast datasets to better understand intellectual disabilities, leading to the creation of large decision-support systems for preventive medicine. By analysing large medical data, can new behavioural cues and biomarkers that can improve early diagnosis be developed? AI and robotics can also be further developed for the treatment of individuals with intellectual disabilities. For instance, a substantial body of research shows how intelligent robots can facilitate therapy for individuals with autism spectrum disorder, which is often associated with intellectual disabilities. Intelligent robots serve as simpler social partners for individuals with ASD, who can more easily be prompted to engage in simulated social interaction with a robot. How can new systems build on these studies to create new social interaction skills, and then generalize to human-human interaction?

The integration of AI technology in healthcare carries the responsibility of ensuring that AI is used ethically and in a way that secures privacy. Biomedical Engineers should prompt machine learning researchers to improve the legibility of AI models so that decisions based on their results are transparent and understandable for practitioners and patients. It is imperative that any technology is designed to complement rather than replace human expertise and empathy. It is critical to provide adequate training for the healthcare workforce to prepare them for effective collaboration with new AI technologies and to understand the implications for roles and relationships within the healthcare environment.

Public funders should prioritize supporting interdisciplinary patient-centred research that aims at creating services and products for individuals with intellectual disabilities. This group often does not represent a significant market for the major multinational corporations are investing heavily in AI and healthcare technologies. It is important to note that the RRI approach mandates that target population must be involved at all stages, from the conceptualization and design of AI technologies. This will mitigate a known limitation of AI models, which are often biased due to the underrepresentation of populations such as those individuals with intellectual disabilities. However, developing AI technology for individuals with intellectual disabilities is more than a matter of equity, inclusion, and dignity; along with these essential benefits, there exists substantial economic potential. By investing in accessible and user-centred technology, we can empower individuals with intellectual disabilities to lead more independent, fulfilling lives and fully participate in society, and simultaneously reduce the costs of healthcare systems and foster the growth of productive individuals.

As we move forward in the AI revolution, it is our collective responsibility to ensure that the benefits of technology reach everyone, regardless of their cognitive abilities. Biomedical Engineers have a critical role to play in ensuring that these technologies respond to this challenge.

